# Editorial: Insights in thalassemia: from genomics to clinical practice

**DOI:** 10.3389/fped.2023.1222946

**Published:** 2023-07-14

**Authors:** Catherine Lynn T. Silao, Zarina Abdul Latiff, Petros Kountouris, Bin Alwi Zilfalil

**Affiliations:** ^1^ Institute of Human Genetics, National Institutes of Health, Manila, Philippines; ^2^ Department of Pediatrics, College of Medicine and Philippine General Hospital, University of the Philippines Manila, Manila, Philippines; ^3^ Department of Paediatrics, Faculty of Medicine, Universiti Kebangsaan Malaysia Medical Centre, Kuala Lumpur, Malaysia; ^4^ Molecular Genetics Thalassaemia Department, The Cyprus Institute of Neurology and Genetics, Nicosia, Cyprus; ^5^ Human Genome Centre, School of Medical Sciences, Universiti Sains Malaysia, Health Campus, Kelantan, Malaysia

**Keywords:** thalassemia, genomics, clinical, quality of life, public health

**Editorial on the Research Topic** Insights in thalassemia: from genomics to clinical practice

Thalassemia, a common inherited autosomal recessive disorder, is characterized by inefficient or absent hemoglobin synthesis, resulting in various severities of anemia (1). Though considered a global medical and public health concern, its greatest impact is clearly felt in countries with limited resources (2). Many aspects still need to be investigated despite the advances and improvements in diagnosis and treatment practices (3).

In some countries, public awareness and health education campaigns, thalassemia registries, prevention programs, improved diagnostics, comprehensive management, and counseling were established to reduce the number of affected births, diagnose cases early, and improve disease management. In developed nations, these needs have largely been satisfied; however, in low-income countries with inadequate access to healthcare, this is still not the case. The main public health strategy for disease control still relies on advanced technology and proper knowledge of the disorder to provide precise screening and diagnosis (2, 4–6). The predictive nature of genetic information, its implications for family members, decision-making, and the associated ethical issues make counseling crucial (7). Therefore, it must be founded on an accurate thalassemia diagnosis using internationally established standards, such as the guidelines of the American College of Medical Genetics and Genomics (ACMG) and the Association for Molecular Pathology (AMP) and their specifications for globin genes developed by the ClinGen Hemoglobinopathy Variant Curation Expert Panel, as well as genetic modifiers of the disease (8–11). In their contributions to this research topic, Hernaningsih et al. Yasin et al. and Ahmadabad et al. reported the unique molecular heterogeneity of both alpha and beta thalassemia in specific geographic regions. Padeniya et al. provided further insight into the genotype-phenotype associations of beta thalassemia. Collectively, these findings allow better understanding of the disease pathogenesis, immediately translating to better treatment for affected patients and their families.

In the 1980's, DNA-based techniques, such as restriction fragment length polymorphism analysis and Southern blotting, were developed to detect specific mutations associated with thalassemia. Amniocentesis and chorionic villus sampling were the next procedures in prenatal diagnostics to emerge (12, 13). The safety and accessibility of blood transfusions and iron chelation therapy with medications like deferoxamine and oral chelators (e.g., deferasirox) were enhanced by developments in blood banking, screening, and compatibility testing. These were adopted as standard of care to treat iron overload brought on by frequent blood transfusions.

Good compliance with these traditional forms of treatment (blood transfusions and iron chelation therapies) allows affected children to progress into adulthood. Unfortunately, these are costly and often traumatic, resulting in difficulties in compliance (14). Mohamed et al. reported that poor adherence to iron chelation was noted among transfusion dependent thalassemia adolescents in low-income families. These have significant implications for clinical management, especially in populations that cannot afford chelating agents.

The identification of compatible donors, conditioning regimens, and supportive care then underwent breakthroughs in hematopoietic stem cell transplantation (HSCT). Improved outcomes and reduced complications were observed as transplantation techniques evolved (15). The concept of gene therapy, which aims to treat the underlying genetic abnormality causing thalassemia, gained popularity during the past two decades. Early initiatives centered on inserting functional genes into the cells of thalassemia patients. Challenges, though, such as costs and long-term safety concerns restrict its application. HSCT is currently the only method that can be promoted to cure thalassemia (16). However, it is essential to stop the occurrence of acute graft vs. host disease (aGVHD), a life-threatening complication commonly seen after allogenic HSCT (17). Huang and Luo have demonstrated that CD4+ T cells may be a potential biomarker for aGVHD in children with transfusion dependent beta thalassemia following HSCT and CD8+ T cells may be a biomarker for severe aGVHD.

Next generation sequencing technology, an accurate, quick, and cost-effective molecular diagnostic technique developed for detecting globin gene variants, subsequently became available at the beginning of the 21st century (18, 19). Its uses, advantages, and limitations as a screening and diagnostic tool were explored by Suhaimi et al. who reported it to be beneficial for the implementation of prevention platforms, carrier identification, and the improvement of genetic counseling and prenatal diagnosis programs.

Later advancements in gene editing technologies, such as CRISPR-Cas9, offered more precise and efficient gene correction strategies. In recent years, clinical trials exploring gene therapies, including gene editing and gene addition, have yielded promising results. Understanding the molecular mechanisms of thalassemia led to the development of targeted treatments aimed at modifying specific disease-related pathways. Small molecules, RNA-based therapies, and gene modulation are some approaches that have shown potential in preclinical and early clinical research (20). Despite these, Zakaria et al. point to limitations such as design difficulties, costs, low transfection efficiency, *in vivo* delivery safety, and ethical concerns.

Thalassemia is a debilitating disease that has significant impact on the patients' quality of life. Transfusion-dependent patients have exhibited pessimism, low self-esteem, low intelligence quotients, and poor academic achievement. Patients suffer due to this chronic illness, which unfortunately places social, psychological, and financial strains on their families (21). Othman et al. reported that though majority of the patients' caregivers reported feeling psychologically well, maladaptive coping strategies were observed in some caregivers of transfusion-dependent patients due to elevated anxiety levels and depression.

The disease is chronic from childhood, therefore, both patients and their families experience challenges that necessitate care interventions and psychosocial support. Parents, particularly mothers who are the primary caregivers, experience moderate-to-severe stress resulting from psychosocial distress and a lack of knowledge of the disease. Fears about the patient's condition and anxieties about the future lead to psychological problems and conflicts. Concerns about how the disease was inherited as well as culpability for the child's illness add to their sense of guilt. Due to frequent medical consultations and the long-term treatment required for the patient, financial support is another significant concern (22). Efforts must, therefore, minimize the suffering of patients and their families through appropriate psychological care, education, counseling, thalassemia support groups, genetic control programs, and support from health authorities. Regardless of the obstacles, it is critical that health professionals from developed and low-income countries network and collaborate to build sustainable, long-term policies and initiatives that improve the quality of life for thalassemia patients and their families.

The articles presented here underscore the importance of managing this disease from a multidisciplinary perspective. Despite the enormous improvements made, there are still many facets of the disease that require attention.
